# iPSC technology-based regenerative medicine for kidney diseases

**DOI:** 10.1007/s10157-021-02030-x

**Published:** 2021-03-03

**Authors:** Kenji Osafune

**Affiliations:** Center for iPS Cell Research and Application (CiRA), Kyoto University, 53 Kawahara-cho, Shogoin, Sakyo-ku, Kyoto, 606-8507 Japan

**Keywords:** iPSC, Kidney regeneration, Nephron progenitor cell, Ureteric bud, Cell therapy, Disease modeling

## Abstract

With few curative treatments for kidney diseases, increasing attention has been paid to regenerative medicine as a new therapeutic option. Recent progress in kidney regeneration using human-induced pluripotent stem cells (hiPSCs) is noteworthy. Based on the knowledge of kidney development, the directed differentiation of hiPSCs into two embryonic kidney progenitors, nephron progenitor cells (NPCs) and ureteric bud (UB), has been established, enabling the generation of nephron and collecting duct organoids. Furthermore, human kidney tissues can be generated from these hiPSC-derived progenitors, in which NPC-derived glomeruli and renal tubules and UB-derived collecting ducts are interconnected. The induced kidney tissues are further vascularized when transplanted into immunodeficient mice. In addition to the kidney reconstruction for use in transplantation, it has been demonstrated that cell therapy using hiPSC-derived NPCs ameliorates acute kidney injury (AKI) in mice. Disease modeling and drug discovery research using disease-specific hiPSCs has also been vigorously conducted for intractable kidney disorders, such as autosomal dominant polycystic kidney disease (ADPKD). In an attempt to address the complications associated with kidney diseases, hiPSC-derived erythropoietin (EPO)-producing cells were successfully generated to discover drugs and develop cell therapy for renal anemia. This review summarizes the current status and future perspectives of developmental biology of kidney and iPSC technology-based regenerative medicine for kidney diseases.

## Introduction

Kidney diseases cause enormous medical problems and economical burden worldwide, but there are few curative treatments except for renal transplantation, which is hampered by severe donor organ shortage [1]. One solution is the development of a regenerative medicine strategy using human pluripotent stem cells (hPSCs), such as embryonic stem cells (hESCs) and induced pluripotent stem cells (hiPSCs) [2]. Because of their potential to infinitely proliferate and to differentiate into any cell type in the body including renal cells, hPSCs are expected to serve as a cell source for regenerative medicine, such as kidney reconstruction and cell therapy. In addition, disease-specific hPSCs that have the genetic predisposition to cause the specific disease can be used to develop models for pathological analysis and drug discovery, in which the injured cell types differentiated from the hPSCs reproduce the disease phenotypes in vitro [2].

In this article, I summarize the recent advances in kidney regeneration research based on developmental biology and describe future perspectives of regenerative medicine and disease modeling for kidney diseases.

## Kidney development

**Fig. 1:**
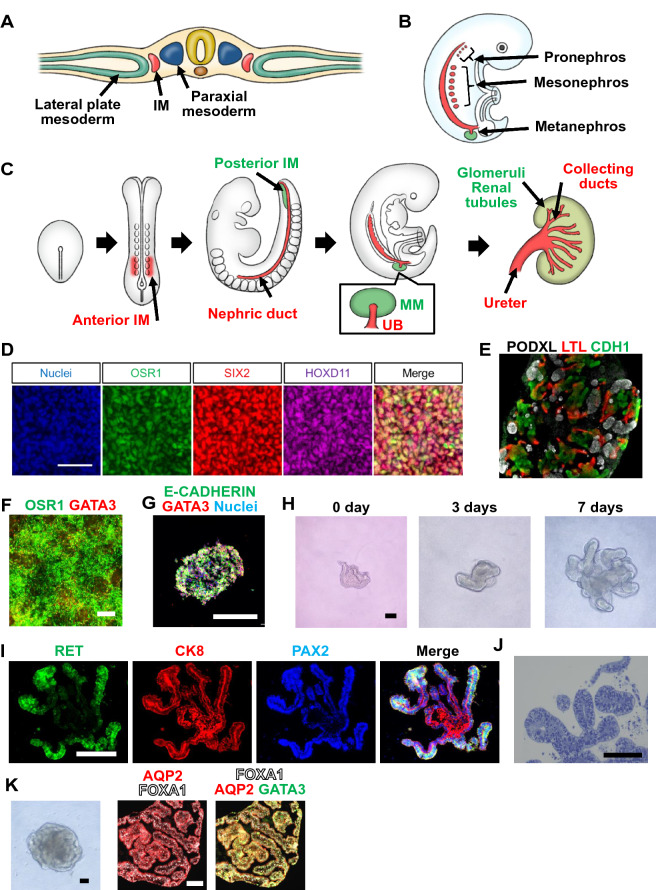
Directed differentiation of kidney lineage cells. **a**-**c** Schematic drawings showing mesoderm specification (**a**), the formation of three kidneys (**b**) and metanephros development (**c**). IM: intermediate mesoderm; MM: metanephric mesenchyme; UB: ureteric bud. **d** Immunostaining of hiPSC-derived nephron progenitor cells (NPCs) for OSR1, SIX2 and HOXD11. **e** Immunostaining of a nephron organoid formed from hiPSC-derived NPCs after 10 days of air–liquid interface culture. PODXL: Podocalyxin (podocyte marker; white); LTL: *Lotus tetragonolobus* lectin (proximal tubule marker; red); CDH1: CADHERIN 1 (distal tubule marker; green). **f**, **g** Immunostaining of anterior IM cells for OSR1 (green) and GATA3 (red; f) and a nephric duct cell aggregate for E-CADHERIN (green), GATA3 (red) and nuclei (blue; g). **h** Morphological change during the reconstruction of branching iUB organoids for 7 days. **i** Immunostaining of an iUB organoid for RET (green), CK8 (red) and PAX2 (blue). **j** Toluidine blue staining of an iUB organoid showing tubular lumens. **k** Bright-field (left) and immunostaining images (middle and right) of collecting duct-like tubular structures derived from an iUB organoid for FOXA1 (white), AQP2 (red) and GATA3 (green). Scale bars, 100 μm. (d) and (e) are adapted from Tsujimoto et al. [12], (f) and (g)–(k) are adapted from Mae et al. [14, 15], respectively

Kidney is derived from an early embryonic germ layer, the intermediate mesoderm (IM) [3] (Fig. 1a). In vertebrates, IM successively gives rise to three kidneys, pronephros, mesonephros and metanephros (Fig. 1b). Mesonephros is the adult kidney of fish and amphibians, while metanephros is the adult kidney of reptiles, birds and mammals. While these three kidneys are similar in that they consist of nephrons, a functional unit of the kidney, their number of nephrons differs. Mammalian adult kidney metanephros is formed by a reciprocal interaction between two IM-derived embryonic tissues, the metanephric mesenchyme (MM) and ureteric bud (UB; Fig. 1c). MM gives rise to the nephrons and interstitium of adult kidneys, while UB differentiates to elaborate the lower urinary tract from the collecting ducts to a part of the urinary bladder [3].

By creating a novel clonogenic assay, we for the first time demonstrated that MM contains multipotent progenitor cells that can differentiate into multiple epithelial cell types constituting nephrons, such as glomerular podocytes and renal tubular epithelia [4]. Later, lineage tracing experiments revealed that these progenitors are marked by the transcription factor Six2 [5]. These progenitors are now called nephron progenitor cells (NPCs). Taguchi et al. demonstrated that IM is divided into anterior and posterior domains, which give rise to UB and MM, respectively [6]. Based on these findings of kidney development, vigorous efforts have been performed to regenerate kidney lineage cells from hPSCs.

### Directed differentiation of hPSCs into kidney lineages

As the first step to directly differentiate hiPSCs into kidney lineages by mimicking kidney development, our group focused on the generation of IM cells and generated reporter hiPSC lines for OSR1 gene, a specific marker for IM [7], by gene editing [8]. Using a quantitative evaluation system and the reporter hiPSC lines, we developed a highly efficient differentiation protocol that induces hiPSCs into OSR1-expressing IM cells. These induced IM cells showed the developmental potential to further differentiate into adult renal cell types, such as glomerular podocytes and renal tubule cells, in vitro and to form three-dimensional (3D) tubular structures by coculturing with mouse metanephric cells [8, 9].

Taguchi et al. for the first time developed selective differentiation methods to induce NPCs through posterior IM from both mouse ESCs (mESCs) and hiPSCs and also generated nephron organoids that contained glomeruli and renal tubules in vitro from the induced NPCs [6]. Takasato et al. reported the generation of kidney organoids that contained multiple renal cell types, such as glomeruli, renal tubules, collecting ducts, stromal cells and vascular cells [10]. By developing a 2D culture system, Morizane et al. efficiently generated NPCs from hiPSCs and then nephron organoids from the NPCs [11]. More recently, our group developed a stepwise differentiation method to efficiently induce hiPSCs into NPCs with the differentiation potential to form nephron organoids [12] (Fig. 1d, e). Our differentiation method which consists of 6 steps more closely recapitulate the natural developmental process of NPCs than the reported methods by Takasato et al. and Morizane et al. [10, 11] and more efficiently generates NPCs in 2D differentiation format than the method by Taguchi et al. which uses 3D culture [6].

Regarding the directed differentiation of UB lineages, Taguchi and Nishinakamura differentiated mESCs and hiPSCs through anterior IM and nephric duct (ND) into UB-like structures [13]. However, the induction efficiency of ND epithelia was low, and the purification of the ND cells by flow cytometry is required for subsequent analyses. We developed a more efficient 2D differentiation method that produces ND epithelia from hiPSCs through anterior IM and includes subsequent differentiation steps without purification [14] (Fig. 1f, g). The induced ND cells formed UB-like structures with RET (+) tip and CK8( +) trunk domains in 3D culture. However, the UB-like structures generated by both groups showed limited branching potential.

More recently, by modifying our UB differentiation method, we successfully generated induced UB (iUB) organoids that possess epithelial polarity, tubular lumens and repeated branching morphogenesis [15] (Fig. 1h–j). Moreover, we succeeded in inducing these iUB organoids to differentiate into collecting duct organoids corresponding to their in vivo counterparts in gestational week 7 human embryos (Fig. 1k).

### Expansion of kidney progenitors

To supply a huge amount of renal cells for basic and clinical research, in vitro expansion culture methods for embryonic kidney progenitors have been investigated. Brown et al. and Tanigawa et al. reported the in vitro expansion of NPCs removed from mouse embryos [16, 17]. Li et al. expanded and maintained NPCs derived from both mouse and human embryos in vitro for a long time using a 3D cell aggregation culture for 17 and 7 months, respectively [18]. The group also expanded NPCs differentiated from hiPSCs in vitro for 2 months using the same methods. Although all these three methods use bone morphogenetic protein (BMP) 7, the role of BMP7 in NPC expansion remains unknown. By screening chemical compounds, we identified a JAK3 inhibitor, TCS21311, as a substitute for BMP7 in the expansion culture developed by Li et al. [18] and revealed a novel inhibitory role of BMP7 in JAK3-STAT3 signaling for NPC expansion [19]. Moreover, the addition of TCS21311 into the expansion culture improved the proliferation rate of both mouse embryo- and hiPSC-derived NPCs.

More recently, we developed an expansion culture for hiPSC-derived UB cells, in which dissociated single cells from iUB organoids proliferate to form colonies expressing UB tip markers [15]. These tip colonies can reconstitute iUB organoids with repeated branching potential, and this reconstitution process can be repeated at least three times.

### Kidney reconstruction

**Fig. 2:**
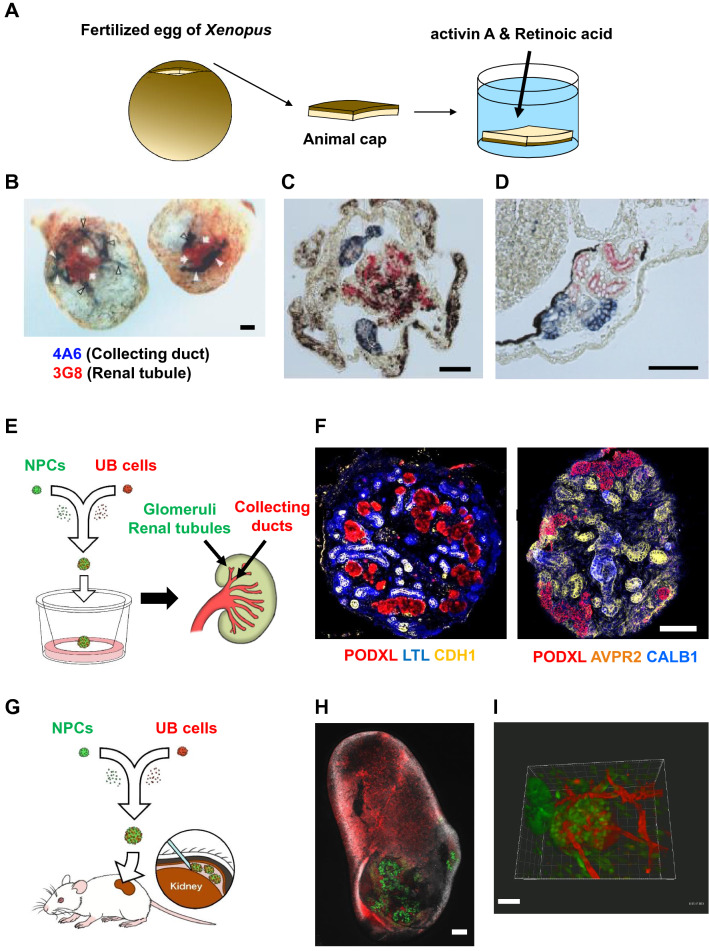
Reconstruction of kidney structures. **a** A schematic showing the generation of pronephros structures from animal cap of *Xenopus* embryos. **b**–**d** Whole mount (**b**) and section double immunostaining images (**c**, **d**) of a stage 42 equivalent *Xenopus* explant (**b**, **c**) and a stage 40 larvae (**d**) using a pronephric tubule-specific antibody (3G8, red) and a pronephric duct-specific antibody (4A6, blue). **e** A schematic showing the in vitro reconstruction of kidney structures from hiPSCs. **f** Triple immunostaining of day 20 kidney organoids for markers of podocytes (PODXL), proximal tubules (LTL) and distal tubules and collecting ducts (CDH1; left), and for PODXL and markers of distal tubules and collecting ducts (AVPR2) and colleting ducts only (CALB1; right). Note that weak CDH1 signals were also found in parts of the LTL^+^ proximal tubules in the left panel. **g** A schematic showing the in vivo reconstruction of kidney structures from hiPSCs. **h** A lower magnification image showing whole host kidney and hiPSC-derived kidney graft (green) after rhodamine B-conjugated dextran administration through the tail vein of host mouse. **i** An intravital multiphoton microscopic image after tail vein injection of rhodamine B-conjugated dextran showing the vessel lumens of host mice penetrate into the hiPSC-derived glomerulus-like structure (green). Scale bars, 100 μm in (b)–(d) and (f), 500 μm in (h) and 40 μm in (i). (b)–(d) and (e)–(i) are adapted from Osafune et al. [22] and Tsujimoto et al. [12], respectively [22]

Earlier works for reconstructing kidney structures used the presumptive ectoderm region of amphibian fertilized eggs, called animal cap, which is a multipotent cell mass (Fig. 2a). Moriya et al. reported that combinatorial treatment with activin A and retinoic acid (RA) induced animal cap to differentiate into pronephric tubules in vitro [20]. Brennan et al. showed that pronephric glomus was also induced in the explants [21]. We demonstrated that the induced explants contained pronephric ducts and that pronephric tissues can be regenerated from amphibian multipotent cells in vitro [22] (Fig. 2b–d). Although the in vitro regeneration system for pronephric kidneys cannot be directly translated to clinical research, it can serve as a simple and useful system for studying kidney development.

In addition to the generation of nephron organoids from mESC- and hPSC-derived NPCs and collecting duct organoids from hPSC-derived UB cells, as described above, Taguchi et al. generated mouse kidney organoids by combining mESC-derived NPCs and UBs and interstitial progenitors removed from mouse embryos, which contain glomeruli, renal tubules and collecting ducts [13]. We generated human kidney organoids in vitro by coculturing NPCs and UB cells that were separately induced from hiPSCs and in which NPC-derived glomeruli and renal tubules and UB-derived collecting ducts are interconnected [12] (Fig. 2e, f). When transplanted into the renal subcapsular space of immunodeficient mice, these hiPSC-derived kidney organoids integrated into the blood vessels of the host mice (Fig. 2g–i).

Regarding the regeneration of kidney organs that have a urinary tract, methods using the experimental animals’ body, such as interspecies blastocyst complementation [23] and organogenic niche method [24], have been investigated. Goto et al. injected wild-type mESCs into the blastocysts of anephric Sall1(− / −) rats and succeeded in interspecifically generating mouse kidneys in the host rat [23]. Fujimoto et al. developed a cell transplantation method through which hiPSC-derived NPCs were transplanted into the kidney development area (i.e. organogenic niche) of mouse embryos in uterine, in which the transplanted and host NPCs together contributed to the chimeric cap mesenchyme, which was connected to the host mouse UB [24]. However, while MM-derived glomeruli and renal tubule were derived from the injected PSCs or NPCs, the remaining kidney-constituent cell types, such as UB-derived collecting ducts and lower urinary tract and vascular cells, were from the host animals in these two strategies.

### Disease modeling

**Fig. 3:**
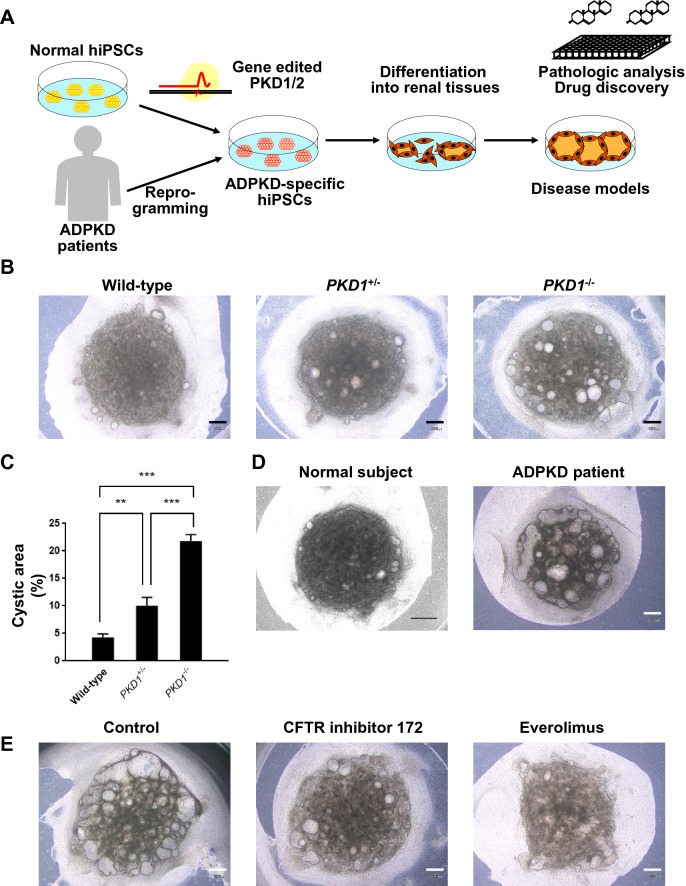
Modeling ADPKD using disease-specific hiPSCs. **a** A schematic showing disease modeling research for ADPKD. Disease-specific hiPSCs are derived by reprogramming the somatic cells of ADPKD patients or gene editing *PKD1/2* in hiPSCs derived from healthy donors. Disease models are generated by differentiating the ADPKD-specific hiPSCs into renal tissues for pathologic analysis and drug discovery. **b** Representative bright-field images of wild-type and gene-edited *PKD1*-mutant hiPSC-derived kidney organoids after 7 days of forskolin treatment. **c** Quantification of the cystic areas of the kidney organoids in (b). Data are represented as the mean ± SE from three independent experiments with four replicates in each. **p < 0.005 and ***p < 0.001 by one-way ANOVA and Bonferroni’s method. **d** Representative bright-field images of normal subject- and ADPKD patient-derived kidney organoids after 7 days of forskolin treatment. **e** Representative bright-field images of patient-derived kidney organoids after 7 days of treatment with CFTR inhibitor 172 (100 μM) or everolimus (10 μM) in the presence of forskolin. Scale bars, 300 μm in (b), (d, right) and (e) and 500 μm in (d, left). Adapted from Shimizu et al. [31]

iPSC technology has made it possible to create in vitro disease models, in which disease-specific hiPSCs derived from patient somatic cells or by editing the causative genes in hiPSCs derived from healthy donors are differentiated into the injured cell types to mimic the disease phenotypes [2] (Fig. 3a). Earlier work by Freedman et al. generated hiPSCs from patients with autosomal dominant polycystic kidney disease (ADPKD), which is caused by mutations in *PKD1* gene, and found that hiPSCs and their differentiated cells showed a downregulation of polycystin 2, elucidating a novel mechanism in which polycystin 1 encoded by *PKD1* gene regulates the expression of polycystin 2 [25]. Our group generated hiPSCs from ADPKD patients, including those complicated with intracranial aneurysms, and confirmed that the vascular cells differentiated from the hiPSCs showed altered intracellular calcium handling and expression of extracellular matrix-related genes, consistent with the vascular cells of ADPKD mouse models and renal cyst cells obtained from ADPKD patients [26].

Recent advances in the generation of nephron organoids from hiPSCs have enabled the modeling of kidney disorders, such as nephronophthisis [27], congenital nephrotic syndrome [28] and autosomal recessive polycystic kidney disease (ARPKD) [29]. Renal cyst models of ADPKD using nephron organoids have been generated from gene-edited homozygous *PKD1/2*-mutant hESCs [30]. However, those models did not recapitulate the renal cyst phenotypes seen in ADPKD when using ADPKD patient-derived or gene-edited heterozygous *PKD1*-mutant hiPSCs. In contrast, we recently generated nephron organoids from both ADPKD patient-derived and gene-edited heterozygous and homozygous *PKD1*-mutant hiPSCs to reproduce renal cyst legions by forskolin treatment [31]. Of note, we confirmed that renal cysts can be formed from all three hiPSC types (Fig. 3b-d). These renal cysts responded to some drugs known to inhibit cyst formation in ADPKD, such as mammalian target of rapamycin (mTOR), indicating that these models can be used to screen drug compounds that prevent renal cyst formation [31] (Fig. 3e). We are currently setting up high-throughput chemical screening systems by modifying the cyst models for therapeutic drug discovery for ADPKD.

### Addressing the complications of kidney diseases

**Fig. 4:**
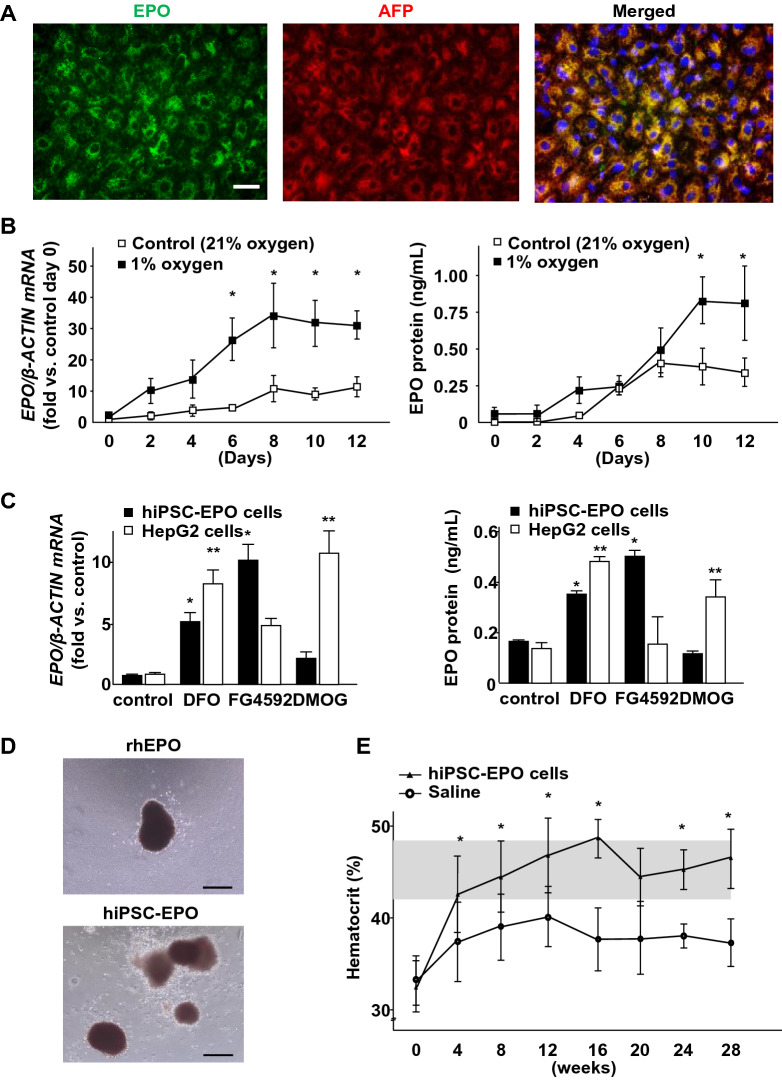
Differentiation of EPO-producing cells and cell therapy against renal anemia. **a** Immunostaining of hiPSC-derived EPO-producing cells (hiPSC-EPO cells) for EPO (green) and AFP (red). **b** Time-course analyses of EPO mRNA expression (left) and protein secretion (right) by hiPSC-EPO cells cultured under low (1%) and normal oxygen (21%) conditions. EPO mRNA expression and protein secretion were analyzed by qRT-PCR and ELISA, respectively. **c** The effects of PHD inhibitors on EPO mRNA expression (left) and protein secretion (right) in HepG2 cells and hiPSC-EPO cells. **d** Representative images of burst-forming unit-erythroid (BFU-E) induced by recombinant human EPO (rhEPO; upper) and hiPSC-EPO protein (lower) in clonogenic hematopoietic progenitor assays using methylcellulose-based semisolid medium. **e** Hematocrit values were evaluated for up to 28 weeks after the transplantation of hiPSC-EPO cells into adenine-induced renal anemia immunodeficient mice (NOD.CB17-*Prkdc*^scid^/J mice). The gray shaded area indicates the normal hematocrit ranges. Scale bars, 40 μm in (a) and 200 μm in (d). Adapted from Hitomi et al. [32]

The major complications associated with chronic kidney disease (CKD) include renal anemia caused by the insufficient production of the hematopoietic hormone erythropoietin (EPO) by the kidneys. Although renal anemia has been successfully treated by the intermittent administration of recombinant human EPO agents, more physiological therapies are required. Considering that EPO is also produced by the liver at embryonic stages or in the case of severe anemia even in adults, we modified a previously reported hepatic differentiation protocol and succeeded in generating EPO-producing cells from hiPSCs (hiPSC-EPO cells) [32] (Fig. 4a). These hiPSC-EPO cells upregulate EPO production in response to hypoxic stimuli, which mimics their in vivo counterparts (Fig. 4b, c). The EPO protein contained in the culture supernatant showed differentiation-promoting effects on erythroid lineages based on a colony-forming assay using human hematopoietic progenitors (Fig. 4d). Furthermore, these hiPSC-EPO cells ameliorated renal anemia for 7 months after transplantation in mouse models induced by adenine administration (Fig. 4e). Thus, hiPSC-EPO cells could be used to discover novel drugs and develop cell therapies against renal anemia [32].

### Cell therapy

**Fig. 5:**
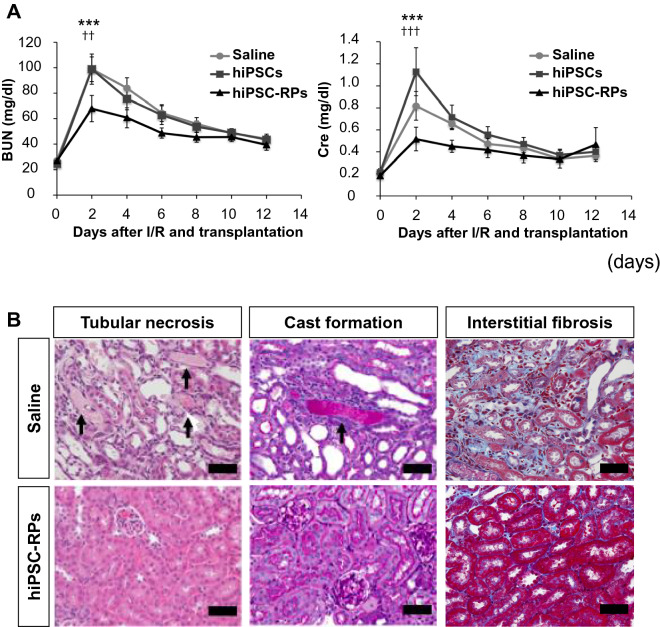
Cell therapy using hiPSC-derived renal progenitors ameliorates acute kidney injury in mice. **a** Time course of blood urea nitrogen (BUN) and plasma creatinine (Cre) levels in acute kidney injury (AKI) mouse models induced by ischemia/reperfusion (I/R) injury that received an injection of saline (circles) or the renal subcapsular transplantation of undifferentiated hiPSCs (hiPSCs, squares) or hiPSC-derived renal progenitors (hiPSC-RPs, triangles). ***p < 0.001 versus saline; ††*p* < 0.01 versus hiPSCs; †††*p* < 0.001 versus hiPSCs. **b** Section images of representative kidney tissue samples from host mice that received an injection of saline (upper panels) or the transplantation of hiPSC-RPs (lower panels) and stained with hematoxylin and eosin (HE, left), periodic acid-Schiff (PAS, middle) or Masson’s trichrome (MT, right) on day 12 after the I/R injury and transplantation. Arrows indicate tubular necrosis in the upper left panel and cast formation in the upper middle panel. Scale bars, 20 μm. Adapted from Toyohara et al. [33]

Research towards cell therapy using hiPSC-derived embryonic kidney progenitors has been carried out. In an attempt to examine their therapeutic potential against kidney diseases, we transplanted NPC-like renal progenitors generated from hiPSCs with our differentiation method into the subcapsule of acute kidney injury (AKI) mouse models induced by ischemia/reperfusion injury, finding the transplantation significantly suppressed the elevation of blood urea nitrogen (BUN) and serum creatinine (Cre) in the host mice [33] (Fig. 5a). Moreover, the treatment significantly ameliorated histological damages caused by AKI, such as tubular necrosis (Fig. 5b). Notably, interstitial fibrosis, which reflects the progression to chronic disease, was significantly prevented as well. The transplanted progenitors ameliorated AKI without being integrated into the host kidney tissues, indicating that paracrine effects by renotrophic factors secreted from the hiPSC-derived renal progenitors are the primary cause of the therapeutic benefits. Elucidating these factors would contribute to developing a cell therapy as well as novel drugs against AKI [33, 34].

Imberti et al. also reported therapeutic benefits of a cell therapy using hiPSC-derived renal progenitors against cisplatin-induced AKI mouse models [35]. They injected hiPSC-derived NPC-like renal progenitors generated with their protocol into AKI mouse models through the tail vein. This transplantation therapy also significantly ameliorated AKI, as evidenced by decreased BUN levels and histological findings.

Although the differentiation protocols to generate the renal progenitors and the AKI mouse models were different, these two reports for the first time demonstrated the potential therapeutic benefits of cell therapy using hiPSC-derived renal progenitors on kidney diseases.

## Conclusion and future perspective

Substantial advances in the generation of embryonic kidney progenitors and kidney tissues from hPSCs have been made. However, there are hurdles to overcome before clinical application. Regarding reconstruction of the kidney, the generation of larger kidney tissues and renal pelvis- and ureter-like structures, into which collecting ducts gather, has not been achieved. In addition, the integration of hiPSC-derived kidney structures to large vessels is required. Cell therapy using hiPSC-derived renal progenitors should also be examined with CKD models. Finally, hiPSC-based models have been developed for some kidney diseases including ADPKD, and the identification of candidate drug compounds against kidney diseases is expected.
